# A Case of Primary Osteomyelitis of the Rib With Involvement of the Chest Wall, Presenting as a Non-Healing Abscess

**DOI:** 10.7759/cureus.26974

**Published:** 2022-07-18

**Authors:** Pardis Abdollahi Zarandi, Suresh Antony, Elsa Sotelo-Rafiq

**Affiliations:** 1 Department of Medicine, Burrell College of Osteopathic Medicine, Las Cruces, USA; 2 Department of Medicine, Las Palmas Medical Center, El Paso, USA; 3 Department of Pathology and Laboratory Medicine, Las Palmas Medical Center, El Paso, USA

**Keywords:** osteomyelitis, chest wall infection, fungal infection, coccidioides immitis, coccidioidomycosis

## Abstract

Coccidioidomycosis of the chest wall is a rare finding and diagnosis is often delayed. We report a case of chest wall abscess with underlying osteomyelitis and an expansile lytic lesion of the left fourth rib in a young and immunocompetent African American male. Initially, the diagnostic assumption gravitated towards bone malignancy, but the diagnosis of coccidioidomycosis was made when the culture results from the bone biopsy specimen confirmed *Coccidioides immitis* as the causative agent. The aim of this unique case is to demonstrate that as an emerging infectious agent, *Coccidioides immitis* is a known cause of chest wall abscess and should be considered among the differential diagnosis by clinicians.

## Introduction

*Coccidioides immitis* is a well-known cause of fungal infection in the Southwest United States where it is endemic, specifically in Texas, New Mexico, Arizona, and California. It is transmitted via inhalation of arthroconidia [1]. Risk factors for the symptomatic and disseminated disease include living in an endemic area, race (African American or Filipino), and male sex [1-3]. Although it typically presents as pulmonary infection in an immunocompromised individual, its uncommon manifestations include chest wall infections and osteomyelitis among others [1-11]. The rarity of such manifestation is of major importance as coccidioidomycosis of the chest wall can disseminate and cause deleterious effects if left untreated, especially in the African American and Filipino population [3].

We present the case of a young and healthy African American male from New Mexico who developed coccidioidomycosis of the chest wall with underlying osteomyelitis without evidence of disseminated disease. This case report illustrates the importance of considering coccidioidomycosis as a rare cause of chest wall infections, especially in endemic regions.

## Case presentation

A 20-year-old immunocompetent African American male with a history of recent unintentional weight loss of 20 pounds presented to the Emergency Department with the complaint of a left breast mass with progressive pain, swelling, and erythema for the past two weeks. He reported a history of pneumonia four months prior to the presentation of current symptoms but no further details on the diagnosis of distant pneumonia were reported. The review of systems was positive for left medial chest wall mass with associated pain, swelling, and erythema, and recent weight loss of 20 lbs. His surgical and family histories were non-contributory. He lived at home with his father and worked as a housekeeper. He denied alcohol and tobacco consumption but reported occasional marijuana use.

**Figure 1:**
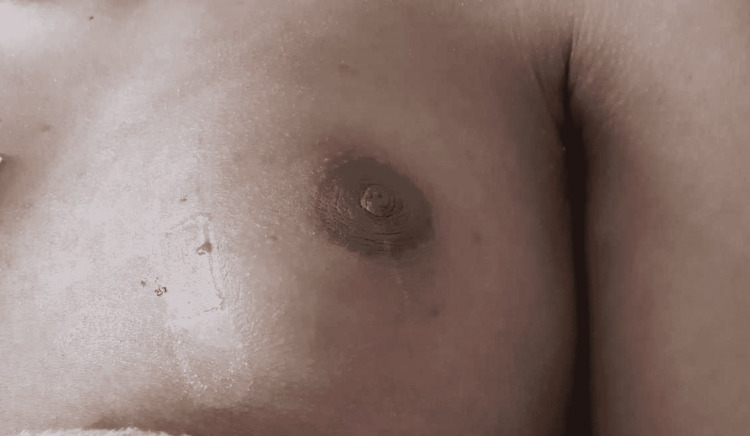
Anterior chest wall area shows moderate swelling and erythema

**Figure 2:**
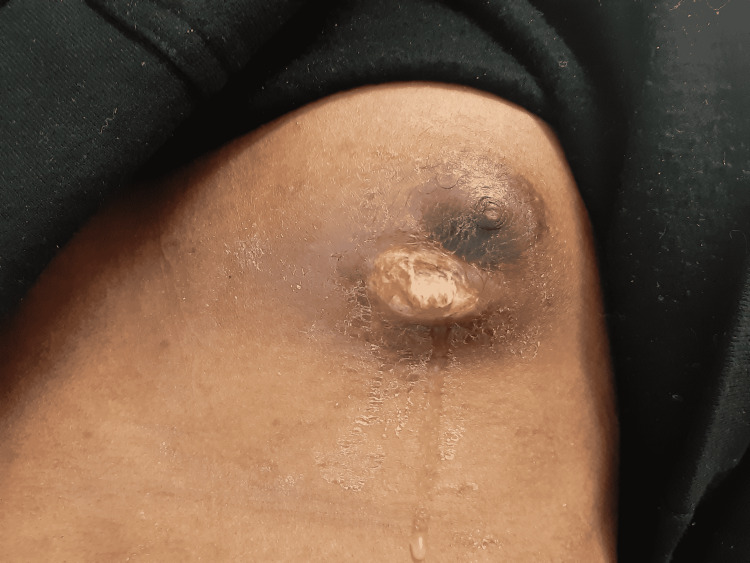
Chest wall lesion with moderate purulent drainage two weeks after discharge from the hospital

Upon initial presentation and during the course of his hospital stay, his vital signs remained within normal range. Physical exam was notable for a mass on the left medial chest wall that exhibited moderate tenderness, erythema, and swelling with a wound that measured about 2 mm. Mild serosanguinous drainage was noted from the wound opening (Figure 1). The lesion increased in size in the span of two weeks from 2 mm to 4 cm and the drainage became purulent and moderate in quantity (Figure 2). No cervical or axillary lymphadenopathy was found on the exam.

**Figure 3:**
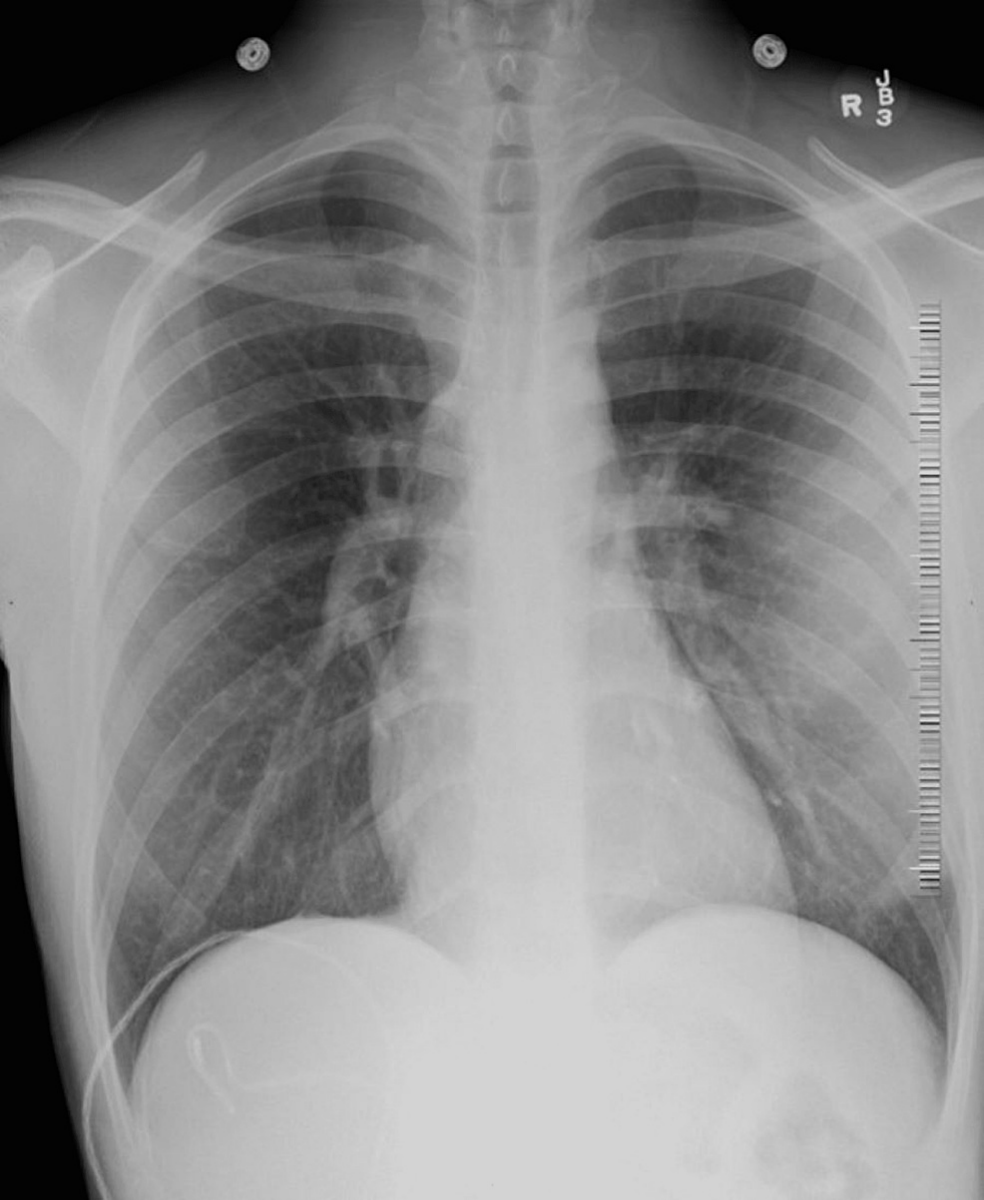
No acute abnormality on the chest X-ray

**Figure 4:**
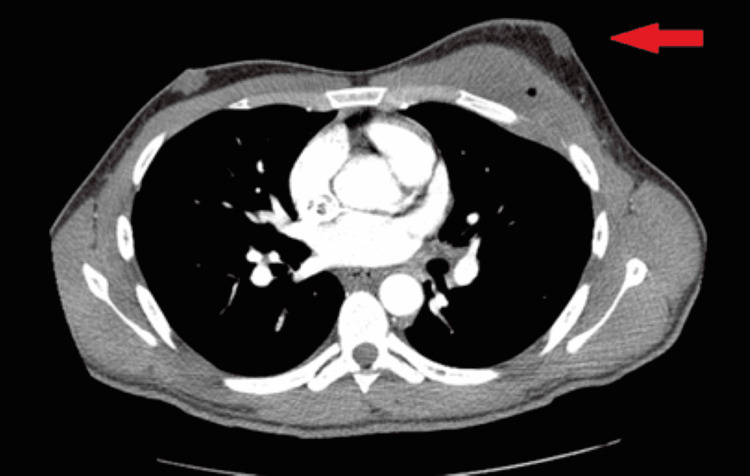
Computed tomography angiography of the chest Phlegmon-like subcutaneous lesion with skin thickening and expansile erosion of the anterior aspect of the left fourth rib.

While in the hospital, white blood cell count (Reference 4.00-10.50 K/mm³) including eosinophil levels remained normal (Reference 0.0-6%). C-Reactive Protein was elevated at 8.1 mg/L (Reference 0.0-0.9 mg/L) according to our laboratory ranges. The remainder of the laboratory results were not significant and HIV status was negative. His chest X-ray did not show any acute abnormality (Figure 3). However, computed tomography angiography (CTA) of the chest with IV contrast was significant for expansile erosion of the anterior aspect of the left fourth rib with encroachment on the left anterior pleural space and infiltration of the left pectoralis muscle (Figure 4).

**Figure 5:**
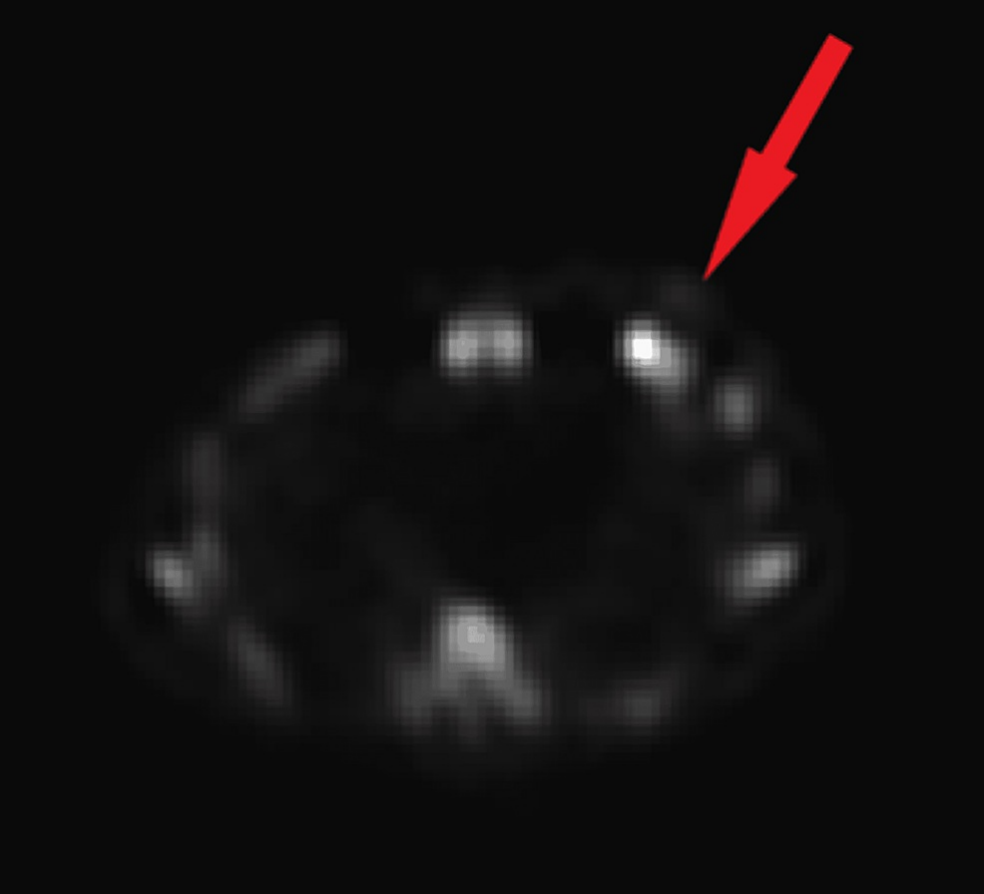
Nuclear medicine bone scan Abnormal uptake of the nuclear tracer in the anterior aspect of left third through fifth ribs.

The initial differential diagnosis included neoplasm versus infectious process. A CT-guided needle biopsy was successfully performed, and the specimen was sent for culture. The preliminary pathology report from the biopsy specimen was notable for granulomatous inflammation with necrosis of the connective tissue with marked acute and chronic inflammation, hemorrhage, and vascular congestion with rare refractile bodies. No malignant cells were found in the specimen. The nuclear medicine bone scan three-phase study displayed increased delayed bone phase radiotracer activity in the anterior aspects of the left third through fifth ribs which corresponds to known fourth rib mass and reactive periosteal changes in adjacent ribs (Figure 5).

**Figure 6:**
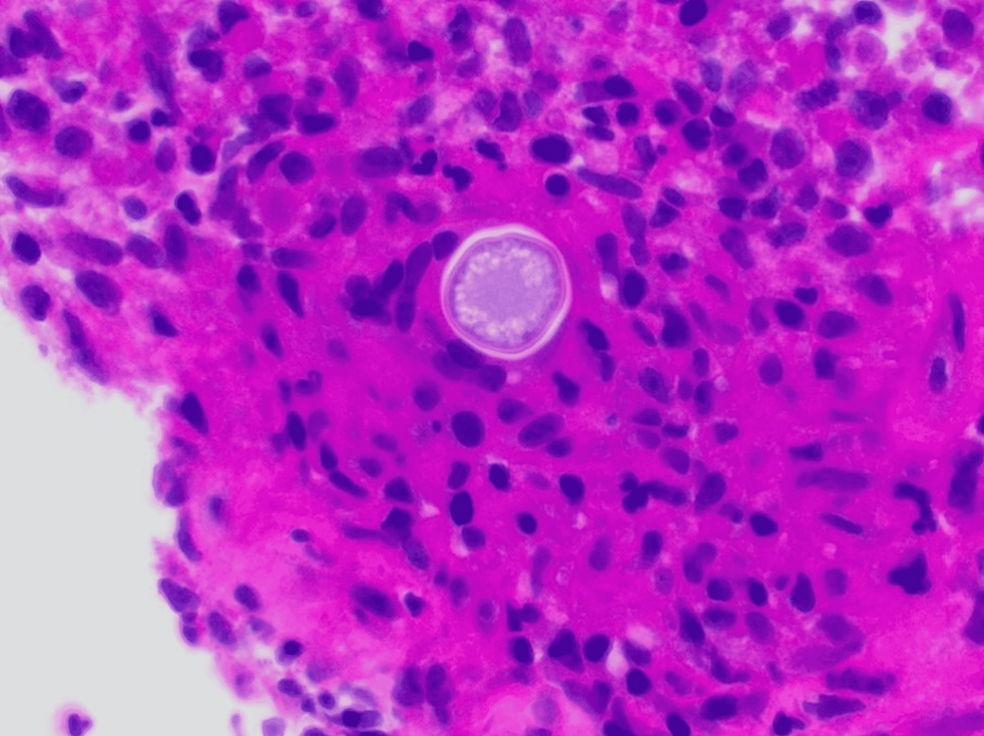
Hematoxylin-Eosin (H&E) stain A large thick-walled spherule containing endospores is characteristic of *Coccidioides immitis*. The spherules are usually found within a granuloma and are surrounded by acute inflammatory cells.

**Figure 7:**
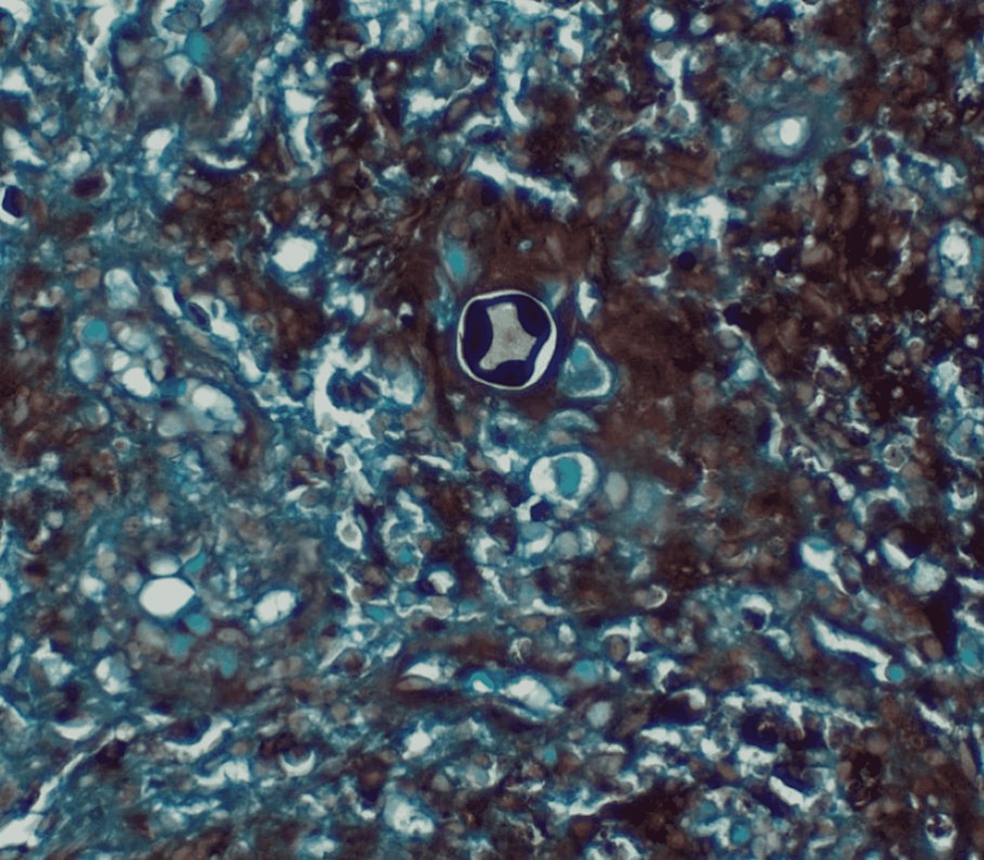
Grocott’s methenamine silver stain Thick-coated spherule is characteristic of *Coccidioides immitis*.

The specimen from the bone biopsy initially grew mold which was isolated and soon identified as *Coccidioides immitis*. A characteristic histological feature of *Coccidioides immitis *noted was the spherules that contain endospores on Hematoxylin-Eosin (H&E) stain. The spherules were circumscribed by a thick wall and held within an aggregation of multinucleated giant cells, forming a granuloma (Figure 6). The fungal organism stains black on Grocott’s Methenamine Stain (GMS) which is one of the best confirmatory stains for *Coccidioides *species [4] that outlined the thick-coated spherule (Figure 7).

The initial inpatient treatment included intravenous Piperacillin-Tazobactam 3.375 grams every 8 hours and Ertapenem 1 gram once daily. The patient was discharged from the hospital before the fungal culture identification results became available. He returned to the clinic two weeks later and was started on 200 mg of oral Fluconazole BID for the first month and one tablet daily lifelong. Upon one month follow-up visit, the patient reported significant resolution of the chest mass with minimal drainage.

## Discussion

Valley fever, also known as coccidioidomycosis, is a fungal infection that primarily affects the lungs, causing pneumonia. This pathogen has the ability to spread to any organ in the body. Usually, patients report a distant episode of pneumonia-like symptoms, with or without arthralgia, before the onset of fungemia and disseminated disease [12]. Most publications have focused on the pulmonary sequela of this infection and its spread to the brain and spinal cord. As high as 60% of individuals are asymptomatic and only about 5% of symptomatic patients and 1% of overall patients develop dissemination [13-15]. It is rare for immunocompetent persons to experience dissemination as it is mostly found in immunocompromised states such as in those infected with HIV, malignancy, or diabetes mellitus and in African American, Filipino, and Hispanic males [1-2].

Although there is no consensus on the treatment of fungal osteomyelitis of the chest wall, the current general recommendations include the long-term use of azole therapy [8,14]. In the past, Amphotericin B was an initial choice for the treatment of disseminated fungal diseases, but its use has been on a downward trend due to its side effect profile which includes renal toxicity and electrolyte abnormality [8,14]. However, if coccidioidomycosis does not respond to azole therapy, liposomal Amphotericin B is sometimes used as an adjunct to surgical debridement [8]. With the introduction of newer treatment recommendations, clinicians now prefer to use azoles due to their lower side effect profile, oral availability, and relatively equal efficacy [14]. The recommended fluconazole therapy for coccidioidomycosis is 200-400 mg PO once a day or Itraconazole 200 mg BID or TID [14]. Surgical debridement is also an available treatment option in patients that do not respond to medical therapy alone. Candidacy for surgical therapy should be evaluated by an experienced surgeon and is usually recommended to patients who are nonresponsive to medication, those with neurological deficits, or intractable pain [14].

A literature review of Medline and PubMed revealed only seven case reports of chest wall infection caused by *Coccidioides immitis* (Table 1). From the seven reported cases, only one was immunocompromised and none of the cases involved the central nervous system. Similarly, the patient in our case report was immunocompetent and did not involve the central nervous system. All the cases were treated with intravenous Amphotericin B and/or Azole therapy. The majority of the case reports mentioned that long-term and/or life-long azole therapy was recommended to prevent recurrence [5-11]. Our patient was treated with 400 mg of Fluconazole and was advised to continue this regimen lifelong.

**Table 1:** Literature review of Medline and PubMed Seven reported cases of chest wall coccidioidomycosis CNS: Central nervous system

Author and year	Age Sex	Race	Immuno- compromised	Site	Imaging	CNS involvement	Treatment	Outcome
Dhital et al., 2021 [5]	33 Male	Hispanic	No	Right chest wall	Chest X-ray: multiple abscesses & fluid cystic lesions	No	Amphotericin for one week. Oral Fluconazole 400 mg	Wound closed after six months
Nassif et al., 2021 [6]	67 Female	unknown	Yes. History of liposarcoma	Right chest	CT chest: right anterior chest mass extending from the pectoralis major muscle into the costochondral junction of the right third rib	No	Oral Fluconazole 400 mg daily	Four-month follow-up: decrease in chest wall mass. Plan to continue fluconazole for another six months
McConnell et al., 2016 [7]	33 Male	African American	No	Left lateral rib and left humerus and history of right chest wall abscess	MRI shoulder: destructive lesion was seen in a left lateral rib extending into the adjacent soft tissues of the left chest wall	No	High dose Amphotericin	Successfully treated but developed recurrence two years later despite suppressive oral antifungal therapy
Homans & Spencer, 2010 [8]	5 Female	Hispanic	No	Right chest wall	CT chest: fluid collection extending from pleural space to chest wall	No	IV Fluconazole 10 mg/kg daily then for 106 days. Substituted with oral Itraconazole 10 mg/kg daily for 13 months	No recurrence after five years
Evans et al., 2010 [9]	36 Male	African American	No	Anterior precordial and left shoulder	CT chest: fluid collection in the sternum and anterior chest wall. Small fluid collections of left acromion	No	Amphotericin	No recurrence at 12 months
Prabhu et al., 2004 [10]	31 Male	Black	No	Ribs, liver, spleen, pelvis, left femur	Chest X-ray: diffuse pulmonary infiltrates.	No	Voriconazole 100 mg b.i.d. po	stable after 10 months of voriconazole therapy.
Franz et al., 1974 [11]	27 Male	unknown	No	Right fourth costochondral junction	Chest X-ray: R middle lobe consolidation	No	50 mg Amphotericin every other day, discharged and given another 1.25 Gm.	Seven-month follow-up negative chest X-ray

Though chest wall infection due to *Coccidioides immitis* is relatively rare, it carries the risk of dissemination and spread to other areas of the body, including the brain, causing a highly fatal coccidioidal meningitis [16]. It is imperative to rapidly diagnose and treat chest wall coccidioidomycosis in order to prevent dissemination to the brain [16].

In a young and immunocompetent individual with no significant medical history, who presents with chest wall infection and underlying osteomyelitis, *Coccidioides immitis* is not the first organism that comes to mind. Therefore, it is crucial to keep this organism in the differential diagnosis when faced with a patient who fits the clinical picture of coccidioidomycosis and has the risk factors [17]. This case demonstrates the clinical presentation, diagnostic findings, and treatment method for disseminated *Coccidioides immitis* in a 20-year-old African American male from an endemic region who presented with symptoms of osteomyelitis of the left chest wall that included pain, swelling, erythema, and purulent discharge. He reported a remote history of pneumonia four months prior to the onset of the chest wall symptoms. It is highly probable that the pneumonia may have been caused by *Coccidioides immitis* that spread to adjacent structures in the chest wall region, causing osteomyelitis of the left fourth rib. The risk factor for the spread included living in an endemic region, African American race, and male sex. His radiological images were significant for expansile anterior fourth rib erosion and subcutaneous fluid collection. A CT-guided needle biopsy confirmed the suspected pathogen.

Extrapulmonary osteomyelitis involving the rib with extension into the chest wall is an uncommon presentation of coccidioidomycosis and should be in the differential diagnosis in the appropriate setting and geographic locations. Clinicians need to have increased awareness of coccidioidomycosis and consider it among the differential diagnosis in individuals with the known risk factors who present with non-healing chest wall abscesses.

## Conclusions

This case report emphasizes several points. Firstly, the importance of prompt diagnosis, clinical presentation, and treatment of coccidioidomycosis to prevent dissemination given the high mortality rate. Secondly, race and sex are risk factors for the development of symptomatic coccidioidomycosis in immunocompetent individuals. African American and Filipino males are at a higher risk of having severe infections with *Coccidioides immitis*. *Coccidioides immitis* should be considered as a possible cause of fungal chest wall infection in the appropriate settings, especially in endemic regions.
